# New Medical Appeal Boards

**Published:** 1920-02-14

**Authors:** 


### NEW MEDICAL APPEAL BOARDS.

In the aftermath of war few things have caused more trouble to officialdom, and anxiety to the general public, than the vexed question of pensions to disabled officers. In many cases the pensions allowed to these officers, most of them members of that unfortunate class—*les nouveaux pauvres*—have been deplorably inadequate.

The temper of the public has been sorely tried by the extravagance of the Government in its various departments, but it will suffer no undue economy in the pensions of those brave men who, in the defence of their country, are now numbered amongst the "halt and the lame." Thus it is with gladness that we note the setting up of new medical appeal boards by the Ministry of Pensions at Chestergate Huts, Regents Park, N.W.1.

Procedure.

The medical appeal board will hear all appeals based on dissatisfaction, with the assessment of the degree of disablement, and will also deal with claims based on deterioration of health since the last board, such claims being deemed to be based on dissatisfaction with the assessment made. Where made after the expiry of a month, claims in the latter category will be dealt with by special machinery which has been set up for the purpose.

The personnel of the appeal boards will consist of a Deputy Commissioner of Medical Services (as a chairman), an appropriate specialist and a medical assessor: in addition the boards will be furnished with all the revelant medical documents, including proceedings of the previous board, and any medical information which the appellant himself may furnish.

Powers Given to the Boards.

The powers given to the board are very considerable, and are almost without precedent if one translates the official jargon of the communication issued by the Ministry of Pensions correctly. The communication reads as follows: "The board will have power to raise, lower, or confirm an assessment, and the decision will be binding for the currency of the previous award, provided that it will not preclude the review of the assessment during that period, and if the officer claims that his condition has become substantially worse since the decision."

At long last we welcome the new appeal boards, and trust that they will discharge their duties towards those who have done so much "Pro Patria." At the same time we believe that very few cases of real failure, to do justice, will be found: our experience is that over-assessments are far more numerous than under-assessments.

